# Editorial: Calcium phosphates of synthetic and natural origin: current status and future challenges

**DOI:** 10.3389/fbioe.2023.1237272

**Published:** 2023-06-16

**Authors:** Liviu Duta, Valentina Grumezescu

**Affiliations:** National Institute for Lasers, Plasma and Radiation Physics, Magurele, Romania

**Keywords:** calcium phosphates (CaPs), bioactive ceramics, biomaterials, regenerative medicine, instructive scaffolds, nanoparticles, drug delivery and targeting, orthopedic implants

Osteoporosis is a disease of the skeletal system which is caused by the metabolic degradation of the osseous matter, resulting from higher bone resorption vs. formation rates (Compston et al., 2019). Typical side effects include the bone joint defects/diseases. These have long been a difficult and frequent clinical problem in medicine and are mainly caused by age, trauma, infections, tumor resection, congenital malformations or hereditary disorders. The current increase in life expectancy and the expanded frequency of bone injuries and diseases are considered the most important causes for the alarmingly large demand for devices dedicated to the orthopedic field (Dorcioman et al., 2023). Tissue engineering is frequently used to tackle and repair these defects/diseases, generating results that are closely resembling the physiological state and which could bypass the obstacles reported for the conventional bone transplantation (Zhao et al., 2021).

The process of repairing fractures generally involves the use of bone grafts and cements, which are fabricated from osteoconductive ceramic materials, among which one can mention calcium phosphates (CaP). Certain materials in the CaP-family are renowned for their excellent ability to induce cells differentiation towards an osteoproductive phenotype (Florian et al., 2019). When such capability is coupled with suitable degradation rates, the prospect to be timely-replaced by the newly-formed bone tissue becomes feasible.

Due to its excellent biocompatibility, high biomineralization capacity, controlled degradation speed and good osteoconductivity, hydroxyapatite (HA) has been one of the most widely used CaPs as a bone substitute and lately as a carrier for local drug delivery in bone. The majority of the clinically-approved HA-based biomaterials and drug carriers are composed of HA particles in the micrometer range (mHA), granules or cements (Niemann et al., 2022). A significant barrier in the use of these materials is that they can only deliver the drugs extracellularly. It was thus demonstrated that this drawback can be overcome when using intracellular targeted drug delivery mediated via nanomaterials (i.e., HA nanoparticles that penetrate cells, nHA). These can improve the efficiency of the treatment by the controlled delivery of drugs to subcellular regions (Cheng et al., 2017). Unfortunately, one important shortcoming related to the clinical use of nHA is still the scarcity of results on its *in vivo* biodistribution after the implantation process (Duta, 2021; Yang et al.).

In their comprehensive study on an innovative nano-drug delivery system, i.e., zoledronic acid-loaded hyaluronic acid/polyethylene glycol/nano-HA particles (HA-PEG-nHAZOL NPs), Xu et al. have demonstrated the effective inhibition of the proliferation rate in the case of three types of human osteosarcoma cell lines (i.e., 143b, HOS, and MG63). It was shown that the fabricated NPs substantially increased the apoptosis related protein expression and tumor cell apoptosis rate (Xu et al.). Furthermore, *in vivo* experiments have evidenced that the local injection of these NPs stimulated both tumor necrosis and apoptosis, along with granulocyte infiltration in blood vessels. These findings should advance the ZOL nano-drug delivery system as a new approach with huge potential for local treatments to inhibit tumor recurrence in clinical therapy (Xu et al.).

Kim et al. reported on their recent developments on the simultaneous drug and cells delivery as a multifunctional substitute for osteoporotic bone tissue regeneration. Thus, a novel system comprising of alginate‒HA hybrid microspheres was advanced. The role of the microspheres was both to enhance osteogenesis and to carry and deliver quercetin, a representative phytoestrogen that regulates the regeneration metabolism of bone tissues for the patients with osteoporosis (Kim et al.). The hydrophobic quercetin was physically adsorbed to the ceramic powder, and the microspheres were demonstrated to continuously release the drug over a long testing period (i.e., 20 weeks). Thus, it was shown that the cells in the hybrid microspheres preserved a good viability, and their osteogenic differentiation behavior was enhanced in the presence of HA. It was therefore concluded that these novel multi-biofunctional hybrid microspheres presented a great potential as drug and cell delivery vehicles for the regeneration of osteoporotic bone tissue at indeterminate defect sites (Kim et al.).

The effect of a phosphoric acid (H_3_PO_4_) solution containing CaP ion clusters (CPICs) on the minimization of the enamel damage during long-term bracket bonding by dissolving the enamel’s surface and promoting its remineralization was recently reported by Kim et. al. It was thus demonstrated that the CPICs-incorporated H_3_PO_4_ solution diminished the adhesive remnant index and maintained the shear bond strength during bracket debonding, regardless of whether thermocycling was performed or type of the resin. Moreover, the etchant containing CPICs was also demonstrated to both remineralize the enamel and boost its microhardness (Kim et. al.).

Considering that most of the mammalian cranial and maxillofacial bones are membranous and of similar embryonic origin, the rat cranial defect model was advanced as an ideal animal model for *in vivo* experiments in the field of bone tissue engineering. Thus, a 5 mm-diameter rat cranial defect was used by Liu et al. as an experimental defect model with the aim to examine the effects of loading different concentrations of metformin (i.e., 0, 250, 500, and 1,000 μM) onto an α-hemihydrate calcium sulfate/nano-HA (α-CSH/nHA) composite. The reported results have demonstrated that the composite material loaded with a concentration of 500 μM of metformin presented the strongest osteoinduction ability, being a promising candidate for the progress of novel techniques to repair various defects of craniofacial bones (Liu et al.).

In a complementary study, clinically-relevant tibial voids in rats were also used with the aim to comparatively study the *in vivo* biodistribution of locally implanted nHA, mHA or a combination of mHA and nHA. It was found that the majority (i.e., >99.9%) of the implanted HA particles, irrespective of their size, remained at the implantation site even after 4 weeks. This was the first longitudinal *in vivo* HA biodistribution study to demonstrate the safe local implantation of nHA particles in bone and therefore their potential use as localized drug delivery systems (Liu et al.).

In the recent decade, the bone tissue engineering has become an essential tool used to treat various bone traumas. Eventhough nano or micro platforms employing HA as a drug delivery carrier were successfully reported, these should be further explored to allow for a safe clinical translation. In this respect, simple or complex HA-binding drug delivery systems could demonstrate their great potential to treat complicated bone tumors.

In summary, this Research Topic contains original research studies that discuss the current status and future challenges of CaPs, with special emphasis on their micro- and nanoparticles form. The Research Topic of interest were devoted, but not limited to, the use of CaPs in a range of different technologies, among which medical implants and drug delivery were the most targeted ones.
